# Bengali translation and characterisation of four cognitive and trait measures for autism spectrum conditions in India

**DOI:** 10.1186/s13229-016-0111-y

**Published:** 2016-12-03

**Authors:** Alokananda Rudra, Jai Ranjan Ram, Tom Loucas, Matthew K. Belmonte, Bhismadev Chakrabarti

**Affiliations:** 1 School of Psychology and Clinical Language Sciences, University of Reading, Reading, UK; 2 Ben Gurion University of the Negev, Beer-Sheva, Israel; 3 Mental Health Foundation, Kolkata, India; 4 The Com DEALL Trust, Bangalore, India; 5 Nottingham Trent University, Nottingham, UK

**Keywords:** Autism, Theory of mind, Central coherence, Perceptual construal, Assessment, Behaviour, Translation, Validity, India, Bengali

## Abstract

**Background:**

Autism is characterised by atypical social-communicative behaviour and restricted range of interests and repetitive behaviours. These features exist in a continuum in the general population. Behavioural measures validated across cultures and languages are required to quantify the dimensional traits of autism in these social and non-social domains. Bengali is the seventh most spoken language in the world. However, there is a serious dearth of data on standard measures of autism-related social and visual cognition in Bengali.

**Methods:**

Bengali translations of two measures related to social-communicative functioning (the Children’s Reading the Mind in the Eyes Test (RMET) and a facial emotion recognition test with stimuli taken from the Karolinska Directed Emotional Faces database), one measure of visual perceptual disembedding (the Embedded Figures Test), and a questionnaire measure (the Children’s Empathy Quotient) were tested in 25 children with autism spectrum conditions (ASC) and 26 control children (mean age = 10.7 years) in Kolkata, India. Group differences were analysed by *t* test and multiple regression (after accounting for potential effects of gender, IQ, and age).

**Results:**

Behavioural and trait measures were associated with group differences in the expected directions: ASC children scored lower on the Children’s Empathy Quotient and the RMET, as well as on facial emotion recognition, but were faster and more accurate on the Embedded Figures Test. Distributional properties of these measures within groups are similar to those reported in Western countries.

**Conclusions:**

These results provide an empirical demonstration of cross-cultural generalisability and applicability of these standard behavioural and trait measures related to autism, in a major world language.

## Background

The atypicalities in social interaction, restricted range of interests or repetitive behaviour, and sensory perceptual dysmodulation that define and characterise autism [1] are expressed in varied degrees in different individuals, making the broad phenotype of autism a spectrum of conditions. DSM-5 accordingly proposes the replacement of the single disorder in favour of the autism spectrum concept, indicating differing grades of severity of one single diagnosis [1].

Autism spectrum conditions (ASC) can be identified as early as 18 months of age [2], and numerous psychological screening and diagnostic tests have been developed to identify ASC. Most of the screening tests include self-report or parent-report questionnaires, while diagnostic measures tend to involve observation within a semi-structured clinical interview setting. The standard screening and diagnostic tools have been developed in English, which often poses a drawback for countries where English is not the primary language. Autism research in South Asia suffers from a lack of availability of well-characterised tasks and questionnaires in local languages, and culture-specific norms. There are some tools that capture neurodevelopmental conditions in India but none are specific to autism. Data on the validity of standardised questionnaires relevant to autism are vital in order to establish a common toolkit for global research and to build research capacity in low-resource settings. In a recent paper, we have taken a first step in this direction by characterising and validating translated versions of standard screening and diagnostic tools for autism (specifically, the Social Communication Disorder Checklist (SCDC), Social Communication Questionnaire (SCQ), Autism Spectrum Quotient (AQ), and the Autism Diagnostic Observation Schedule (ADOS)) [3].

Behavioural/performance measures constitute the next step in this sequence to characterise the autistic phenotype. These involve direct participation of the individual under study and so complement questionnaire- or interview-based measures. The data from these tasks tend to be continuously distributed, which make them good candidates for the proposed dimensional approach of studying psychopathology using the Research Domain Criteria (RDoC) framework [4], across categorical diagnostic boundaries.

The specific measures of interest in this study relate to core domains in which atypicalities have been observed in ASC, namely social-emotional function and perceptual/low-level sensory function.

The Reading the Mind in the Eyes Test (RMET) is one of the best validated measures of emotion recognition from limited facial cues (eye region only) and is associated with deficits in autism [5]. While it has been translated into several languages worldwide, no data are available on this task in South Asia. The original task was developed for adult participants [6], but a modified version adapted for children has shown similar group differences between autism and neurotypicals [5]. Several of the emotions depicted in the RMET are complex and can be associated with cultural differences in recognition. Thus, it is vital to test the cultural generalisability of the data obtained from this task. The second task related to social-emotional function is a basic emotion recognition task using stimuli from the Karolinska Directed Emotional Faces (KDEF) database [7]. A version of this task has been used in adults and shown to be associated with clear differences between individuals with and without ASC [8]. While cultural differences in this task are not expected due to near-universality of basic emotion expressions, all stimuli in this task are faces of white Caucasian actors. Emotion recognition is influenced by the ethnicity of the stimuli [9, 10] (but can be robust to some differences [11], especially given cultural exposure [12]), and hence, it is of particular interest to test children with and without ASC from a non-white ethnic background with limited exposure to other ethnicities on this task. The third measure related to social-emotional functioning is the child version of the Empathy Quotient (EQ-C), which has been associated with robust group differences between children with and without ASC [9]. EQ-C has been translated to five languages in Europe and America, but the lack of availability of this instrument in any South Asian language limits its comparability and generalisability.

To measure an aspect of sensory perceptual functioning, we administered the Embedded Figures Test (EFT) [10], a measure of perceptual disembedding or, equivalently, field independence or level of construal. Individuals with ASC perform faster and more accurately than matched controls in this task [11–14]. Cultural differences in visual perceptual tasks have been noted in both autism spectrum [15] and typical [16, 17–21] populations and are reflected in cognitive neurophysiology [22, 23]. The aim of the current study was thus to translate into Bengali this battery of four measures related to key autism symptom domains and to characterise them in a sample of children from Kolkata, India, with and without ASC. Bengali is spoken by more than 193 million people as a first or second language around the world, with the majority of speakers in India and Bangladesh (www.ethnologue.com). Characterising these tools in India can indicate the degree to which the behavioural profile of children with or without autism is similar or variable across languages and cultures and will create a vital resource for future research in autism in low-resource settings of South Asia.

## Methods

### Translation

Translators who were bilingual (fluent in English and Bengali) with expertise in autism were identified and approached and explained the nature of the study. Translation was followed by blind back-translation into English by individuals fluent in English and Bengali. This back-translated English version was then matched with the original English version by two bilingual expert raters (AR and BC). This procedure was iterated until rater consensus was reached regarding the usability of the back-translated Bengali version.

### Participants

Twenty-five ASC children aged 9 to 11 years were recruited from multiple clinics in Kolkata. All of them had a certified diagnosis of an autism spectrum disorder (Asperger syndrome or high-functioning autism) using DSM-IV TR criteria from a recognised clinician and confirmed by a clinician-administered ADOS. Furthermore, all ASC children scored above 76 on the Autism Spectrum Quotient: Children’s Version (AQ-C). A score of 76 or above on AQ-C is indicative of ASD [24] and thus provided a further confirmation of the diagnostic status. All ASC children attended schools or special education centres that cater to students from middle or low-middle socio-economic status (SES). Control children were recruited from two mainstream schools: a co-educational school with children of low-middle SES and a girls’ school with children of middle SES (see Table 1 for demographics). The choice of these schools was governed by the prior familiarity of the experimenter. The inclusion criterion for cases was a confirmed diagnosis of ASC, while that for the controls was the absence of any mental health condition, as reported by the parents. Both groups were matched for gender. However, the limited availability of high-functioning autism cases in this age group who were willing to take part in research did not allow us to match the two groups for age or intelligence quotient (IQ).

**Table 1:** Participant demographics

Participant	Gender	Number	Mean age (s.d.)	Mean verbal IQ (s.d.)
ASC	Male	20	11.56 (1.84)	87.8 (2.1)
	Female	5	11.60 (2.21)	88.4 (1.9)
Control	Male	21	9.89 (0.96)	102.0 (4.6)
	Female	5	10.01 (1.3)	95.8 (1.6)

### Tools

The EQ-C, a short parent-report questionnaire comprising 27 items [9], is intended to measure how easily one can pick up on other people’s feelings and how strongly one is affected by them. Children scoring less than 30 show difficulties in identifying and/or responding appropriately to others’ emotions. For the EQ-C, a ‘slightly agree’ or ‘slightly disagree’ response endorsing an empathic trait or behaviour scores one point, ‘definitely agree’ or ‘definitely disagree’ endorsing an empathic trait or behaviour scores two points, and all responses not endorsing empathic behaviour score zero. The maximum possible score is 54. The EQ-C has shown high test–retest reliability, with an intra-class correlation coefficient of 0.86, (*p* < 0.001) [9].

The AQ-C was administered solely to confirm the clinical diagnosis of the ASC participants. The AQ-C is a parent-report questionnaire comprising 50 questions quantifying autistic traits in 4- to 11-year-olds. Children with ASC score high on the AQ-C as compared to typically developing children [24]. The translated and validated version of the AQ-C in Bengali was used for this study [3]. The EQ-C and AQ-C were filled in by the parents of the children selected for the study.

Three behavioural measures were applied:

1. The RMET-Child [5] comprises 28 images of the eye region of the face, each representing a mental state. Each picture is flanked by four words as candidate labels for the depicted emotion. The participant has to indicate the correct answer by pointing. During the process of iterative translation/back-translation, a few of the option words were translated into short phrases (rather than single words) to allow for easy understanding in everyday language. A direct dictionary-based translation from English in such cases would often result in complex words that are not typically used. In order to make the test materials as closely matched to those used worldwide, no stimulus was discarded from the RMET. The RMET stimuli contain just the eye region and not the entire face and are presented in black and white—which can potentially reduce the impact of the ethnicity-related differences that influence emotion perception.
2. The KDEF database, developed in 1998 by Daniel Lundqvist and colleagues in Stockholm (http://www.emotionlab.se/resources/kdef), consists of photographs of facial expressions of basic emotions. In the task based on these stimuli, participants choose amongst seven basic emotion words written below each picture (happy, sad, angry, surprised, disgusted, afraid, and neutral). An online version of this task demonstrates differences in performance between individuals with and without ASC [8]. Both the RMET-Child and the KDEF were printed on A4-sized laminated paper. Twenty-eight faces from the KDEF database (4 faces for each of 7 emotions) were selected. The KDEF stimuli were presented in black and white for reasons described above.
  Before beginning the KDEF and RMET-C tests, the children were provided with a glossary containing all the words/phrases in Bengali. They were asked to familiarise themselves with the words and were given explanations where necessary. The pictures for the RMET-Child and the KDEF were shown to the children one by one. Participants were told to identify the emotion and to say or point to the correct answer with either their index finger or a pen as they preferred. No time limit was imposed.
3. The standard child Embedded Figures Test (EFT) [10] consists of pictures of complex shapes, each of which contains a target simple shape. Twenty-five EFT images were printed and laminated on photographic paper. A fixed set of simple patterns was shown and explained prior to the test. The experimenter first showed the complex design for 15 s, asking the participants to describe it, then revealed the simple shape for 10 s. Then the complex design was presented again and a stopwatch started immediately. The participant was told to look for the simple shape in the complex figure and to trace its lines with their finger or a pencil. Each child was given 1 min to identify and to trace the simple pattern and the response time was noted (as soon as the tracing was completed). The designs were presented in a fixed order, beginning with a practice item followed by the 25 test items. Subjects were encouraged with positive comments if they failed to identify in the first minute. A wrong answer or a ‘pass’ by the participant was taken as an indicator to move on to the next image. The instructions were given in simple Bengali sentences. Responses were recorded by the experimenter and coded for accuracy (if the correct shape was traced). Reaction time was noted using a stopwatch.

All subjects were tested either in their homes or in a quiet room in the clinic or special school. All data were collected in 2013–2014.

Since compliance with a lengthy testing regimen would have been difficult for many subjects with autism (the total administration time for Malin’s Intelligence Scale for Children (MISIC) is 2.5 h on average), only the verbal subtests of MISIC, the Indian adaptation of the Wechsler Intelligence Scale for Children [25], were administered prior to the experiment.

All the tests were administered in person in less than 2 h. Parents of both cases and controls were administered the paper versions of the EQ-C and AQ-C.

### Analysis

Mean scores of the cases and controls were calculated for each tool, separately for each gender. Simple effects of group were assayed by *t* test. In light of significant deviations from normal distribution, Spearman’s correlation coefficients were calculated between all measurements. Multiple regression for each of the dependent measures tested effects of group, gender, age, and verbal IQ.

## Results

Twenty-five ASC children and 26 controls participated. Of the ASC participants, 25.5% attended special schools, 23.5% inclusive schools, and the rest attended special education centres. All control children attended mainstream schools. Mean age for the ASC group was 11.57 (S.D. = 1.8), for the control group 9.92 (S.D. = 1.1). The range of IQ scores for the controls and cases was between 80 and 120. The mean verbal IQ for cases was 87.9 (S.D. = 2.04) and for controls was 100.8 (S.D. = 4.8). Cases and controls were gender matched (Table 1).

**Fig. 1:**
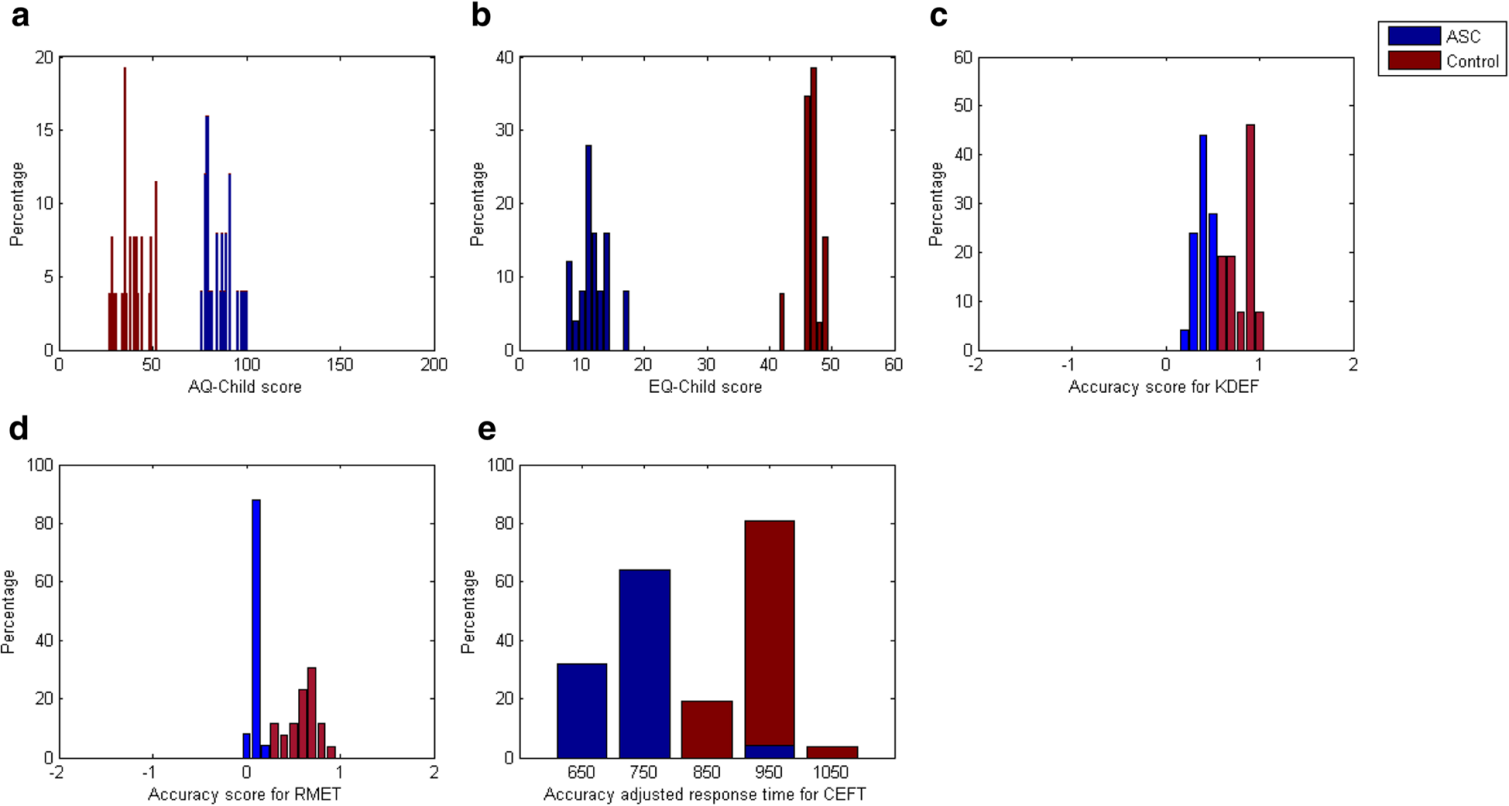
The top panels (**a**–**c**) show the distribution of raw scores for AQ, EQ, and KDEF for cases and controls. The bottom panels (**d**, **e**) show the distribution of raw scores for RMET-C and CEFT for cases and controls

Table 2 shows the mean scores obtained by cases and controls on AQ, EQ, KDEF, and the Child Eyes test and the accuracy-adjusted response time on the Child-EFT (CEFT), separately for each gender. All ASC participants and none of the controls scored above the cut-off of 76 on AQ-C. ASC and control groups differed highly significantly (*p* < 0.001) on all measures (data presented in Table 3). Figure 1 shows the distribution of scores received on all the measures.

**Table 2:** Mean scores obtained by cases and controls on AQ, EQ, KDEF, RMET-Child, and the accuracy-adjusted response time on CEFT according to gender

Behavioural measures	ASC				Control			
	Male		Female		Male		Female	
	Mean	S.D.	Mean	S.D.	Mean	S.D.	Mean	S.D.
Accuracy (Child Eyes test)	0.09	0.03	0.12	0.03	0.58	0.15	0.68	0.1
Accuracy (KDEF)	0.38	0.07	0.41	0.09	0.82	0.12	0.81	0.05
Accuracy (CEFT)	0.78	0.04	0.78	0.04	0.76	0.02	0.75	0.01
Accuracy-adjusted response time (CEFT)	688.23	79.21	683.17	34	973.09	50.4	985.6	53.2
Child-AQ score	85.30	7.6	87.8	5.02	39.67	7.9	39	6.9
Child-EQ score	11.35	2.3	13.40	2.3	46.62	1.9	46.6	0.9

Accuracy is calculated as the total number of correct responses divided by the total number of items. Accuracy-adjusted response time is calculated as the reaction time for a correct response, divided by accuracy

**Table 3:** Group mean scores on each measure and *t* test statistics for the comparison between cases and controls

Cognitive measures	Mean (S.D.)		*t* test	Effect size Cohen’s d
	ASC	Control		
RMET-C (accuracy)	0.09 (0.04)	0.6 (0.15)	16.5***	4.6
KDEF (accuracy)	0.39 (0.07)	0.82 (0.11)	16.3***	4.7
CEFT (RT for correct responses)	687.26 (71.9)	975.49 (50.11)	16.7***	4.7
Child-AQ	85.8 (7.14)	39.54 (7.5)	22.4***	6.3
Child-EQ	11.76 (2.4)	46.62 (1.7)	60.01***	16.8

***Denotes significance at *p* < 0.001

### Correlations between measurements

Spearman’s rank order correlation was computed as the data were not normally distributed. Across the whole sample, the scores on the EQ-C were negatively correlated with that on the AQ-C (Spearman’s rho = −0.776, *p* < 0.001). The EQ-C scores significantly positively correlated with the total accuracy score for the KDEF (Spearman’s rho = 0.780) and the RMET-Child (Spearman’s rho = 0.829), both *p* < 0.001.

Within the control group, EQ-C scores correlated negatively with AQ-C (Spearman’s rho = −0.397, *p* = 0.045) and positively with total accuracy for the KDEF (Spearman’s rho = 0.411, *p* = 0.037). RMET correlated with EQ-C scores (Spearman’s rho = 0.224, *p* = 0.272).

Within the ASC group, EQ-C was not significantly correlated with the AQ-C (Spearman’s rho = 0.271, *p* = 0.190), the KDEF (Spearman’s rho = −0.273, *p* = 0.186), or the RMET-Child (Spearman’s rho = 0.354, *p* = 0.083).

### Multiple regression analysis with gender, age, and IQ as predictors

As the groups differed in IQ and age, a multiple regression analysis was carried out (Table 4). This analysis showed a significant effect of group for each of the measures after accounting for potential effects of age and IQ (RMET-C accuracy scores: *β* = 0.956 *p* < 0.001; CEFT accuracy-adjusted reaction time scores: *β* = 0.958, *p* < 0.001; KDEF accuracy scores: *β* = 0.870, *p* < 0.001; EQ-C: *β* = 0.955, *p* < 0.001). There was no significant interaction between group*verbal IQ, group*age, or age*verbal IQ. To avoid common confounds associated with ANCOVA [26, 27], these analyses were re-run without including IQ and age in the model. No significant changes in the test statistics were observed.

**Table 4:** Multiple regression analysis to test the effect of effect of group, gender, age, and verbal IQ on all measures

Predictors	RMET-C total accuracy score		KDEF total accuracy score		CEFT total response time for correct responses		Child-AQ		Child-EQ	
	*β*	*p*	*β*	*p*	*β*	*p*	*β*	*p*	*β*	*p*
Group	0.956	<0.001	0.870	<0.001	0.958	<0.001	0.904	<0.001	0.955	<0.001
Gender	0.091	0.128	0.025	0.683	0.003	0.964	0.007	0.877	0.027	0.112
Age	0.009	0.894	0.044	0.512	0.088	0.178	0.017	0.328	0.037	0.053
Verbal IQ	0.035	0.772	0.082	0.508	0.091	0.447	0.049	0.600	0.689	0.494

## Discussion

We tested Bengali translations of four trait and behavioural measures related to autism in a sample of children in Kolkata, India. The measures pertaining to socio-emotional functioning include two behavioural tests: complex facial emotion recognition using the ‘Reading the Mind in the Eyes Test’-Child Version (RMET-C) and a basic facial emotion recognition task using stimuli from the Karolinska Directed Emotional Faces (KDEF) database. The trait measure related to social cognition was the Children’s Empathy Quotient (EQ-C) [9]. The behavioural test related to sensory perceptual functioning was the Child-EFT. All of these tools showed group differences between ASC and controls in the expected direction, similar to those in the original reports. These results thus provide evidence for generalisability for these tools and suggest their usability for research in people speaking Bengali (>193 million).

Children with ASC responded significantly less accurately in RMET-C scores compared to the control children. Notably, the maximum number of correct answers obtained by ASC participants was 4 out of 29. This is not significantly higher than chance [5]. In contrast, the controls in our study had an accuracy of 60%, similar to that reported in the original study in the same age range [5]. Similar to the Persian version [28], and in conformity with a sign test of published studies of the original English and translated RMET versions in which no study’s male mean RMET score exceeded its female mean RMET score [29], females scored higher than males on the RMET. The low verbal IQ in the ASC group in our sample may account for the low accuracy in the RMET-C: the ASC group may have found it difficult to identify complex verbal phrases defining emotions. In contrast to the RMET-C, the emotion words in the basic emotion recognition task are simpler, and hence, the ASC group performed comparatively better on this task than on the RMET-C.

It is known that people from East Asia, who are more often studied than South Asian populations, are more prone than are North Americans to explain behaviour using social contexts rather than individuals’ internal states [30, 31]. Furthermore, RMET scores of people born in East Asia are lower than those of people born in North America [32], reinforcing the result that attention to context impairs the detail-orientated analysis necessary to recognise social and other visual information from still pictures [33]. At least a portion of the difference in RMET scores between languages and cultures may stem not from artefacts of languages and translations but actually from real differences in the ways in which visual attention is allocated within social (and non-social) scenes. In this regard, differences in psychological distance and associated level of construal [34] might describe not only cognitive and perceptual differences associated with short-term social psychological manipulations but also long-term effects of culture and biological and cultural effects associated with sex and gender [35].

Similar to the RMET-C, the KDEF task accuracy differed significantly between the ASC and control groups (Table 2). This result is similar to those previously reported by Sucksmith et al. [8] in an adult sample. Most ASC children were able to recognise the ‘happy’ emotion more accurately than negative emotions, as has been previously observed in adults [36]. Similar to this study, deficits in emotion recognition have also been seen between ASC and controls on negative basic emotions in a study by Ashwin et al. on male adults [37]. However, it is possible that this observed lack of differences for recognition of non-negative emotions is driven by the inclusion of just one positive emotion (happy) in the set of emotions tested. The similarity of our results with those obtained in Caucasian samples suggests that the emotion recognition performance, and group differences thereof as seen in ASC, are relatively unaffected by the ethnicity of the actors.

On the EQ-C, ASC children scored considerably lower than the control sample (ASC mean score = 11.76, control mean score = 46.62) (see Table 2). These scores are comparable to those reported in the original British sample (ASC 13.97, S.D. 6.82; controls 37.7, S.D. 9.81) [13]. The observed pattern of sex differences in the scores for both cases and controls also replicated those reported in the original report.

EQ-C scores showed the expected pattern of correlations in the whole sample, i.e. low EQ-C scorers were less accurate in identifying emotions on the KDEF and the RMET-C. A positive correlation between EQ-C and RMET-C has been previously noted in a study of 6- to 9-year-old children [38]. Total EQ-C scores were significantly negatively correlated with the total AQ-C scores for the whole sample. EQ-C scores in controls were significantly negatively correlated to AQ-C, also replicating previous results in adults [6]. Interestingly, however, this pattern of correlations was not observed within the ASC group. This lack of covariation could reflect the limited variation within this group of higher-functioning children with ASC.

In contrast to measures of emotion recognition and empathy, tasks of sensory perceptual function have often been associated with an advantage in individuals with ASC [39, 40]. On the EFT, ASC children are typically faster in detecting simple patterns in complex figures [13]. As predicted, the total response time for ASC children on the CEFT was lower than that for controls, replicating previous results [41, 42]. However, there was no significant difference in the total mean accuracy score between cases and controls, again replicating previous results [12].

Limited resources precluded confirmation of clinical diagnoses by a psychiatrist or with an additional research ADOS administration. However, all the ASC participants had certified diagnostic reports based on DSM-IV (TR)/ICD-10 from recognised clinicians and had a suprathreshold score on the AQ-C. A second limitation is that time constraints precluded measurement of full-scale IQ, which was instead estimated by adding 6% to the verbal test quotients as suggested in the MISIC manual [25]. Third, the verbal component of two of the tasks (KDEF and RMET-C) meant that we could only test high-functioning verbal ASD children. Future studies should develop tasks that can be used in children with a wide range of verbal abilities, including nonverbal children with ASC. Finally, since the participant pool was drawn from mainstream and special schools that serve a largely middle SES urban population, it is difficult to extrapolate these results to the extreme low end of SES or to rural populations. Future studies should attempt to address these limitations by more complete phenotyping in larger, more representative samples.

## Conclusions

We translated and tested three behavioural measures and one trait measure related to various aspects of autistic cognition in Bengali-speaking children in Kolkata, India. We find strong evidence of cultural generalisability for these tools, as well as for group differences between ASC and controls. These results provide the first evidence for the usability of these tools in a major regional language of South Asia and constitute a vital step to standardise measurement of autism-related symptomatology globally.

## Abbreviations

AQ-C: Autism Spectrum Quotient: Children’s Version
ASC: Autism spectrum conditions
EFT: Embedded Figures Test
EQ-C: Children’s Empathy Quotient
IQ: Intelligence quotient
KDEF: Karolinska Directed Emotional Faces (database)
SES: Socio-economic status
